# COVID-19 vaccine development: What lessons can we learn from TB?

**DOI:** 10.1186/s12941-020-00402-x

**Published:** 2020-11-30

**Authors:** Hussain A. Safar, Abu Salim Mustafa, Timothy D. McHugh

**Affiliations:** 1 Centre for Clinical Microbiology, Division of Infection and Immunity, University College London, London, UK; 2 Department of Microbiology, Faculty of Medicine, Kuwait University, Kuwait City, Kuwait

**Keywords:** COVID-19, SARS-CoV-2, Tuberculosis, Vaccine

## Abstract

At the time of writing, the SARS-CoV-2 virus has infected more than 49 million people causing more than 1.2 million deaths worldwide since its emergence from Wuhan, China in December 2019. Vaccine development against SARS-CoV-2 has drawn the global attention in order to stop the spread of the virus, with more than 10 vaccines being tested in phase III clinical trials, as of November 2020. However, critical to vaccine development is consideration of the immunological response elicited as well as biological features of the vaccine and both need to be evaluated thoroughly. Tuberculosis is also a major infectious respiratory disease of worldwide prevalence and the vaccine development for tuberculosis has been ongoing for decades. In this review, we highlight some of the common features, challenges and complications in tuberculosis vaccine development, which may also be relevant for, and inform, COVID-19 vaccine development.

## COVID-19 and TB situation worldwide

The coronavirus disease 2019 (COVID-19) and tuberculosis (TB) are among the major health problems that pose major threats to international public health, both are respiratory infections. The number of severe acute respiratory syndrome coronavirus 2 (SARS-CoV-2) infections has increased dramatically worldwide since its emergence from Wuhan, China in December 2019, with more than 49.7 million confirmed cases in 216 countries, and a worldwide average mortality rate of 2.4% at the time of writing [1], about one quarter of the world’s population is infected with *Mycobacterium tuberculosis*, with 10 million people developing active disease and 1.5 million people dying of TB annually; making TB the leading cause of death from a single infectious agent before the emergence of SARS-CoV-2 [2, 3]. The World Health Organisation (WHO) declared the coronavirus disease COVID-19 as a pandemic in March 2020, however in 1993, TB was declared as a “global emergency” and later in 2016 the WHO developed the “Stop TB Strategy” aiming for a TB-free world in partnership with the “End TB Strategy” 2016–2035 [4]. Vaccine development is a crucial strategy for the prevention of widespread infectious diseases, reducing the morbidity and mortality rates. Long-lasting vaccines can be more cost-effective than chemotherapy and therefore can have a compelling impact on global health, as demonstrated by the successful smallpox and polio vaccines [5]. Even though there is an urgent need to develop an effective vaccine against COVID-19, the immunological and biological features of the COVID-19 vaccine must be evaluated to inform this development and introduction. TB vaccine development has been ongoing for decades exploring numerous approaches and platforms. Here we highlight some of the common features in TB vaccine progress that may contribute to the thinking in COVID-19 vaccine development.

## COVID-19 mechanism of transmission and pathogenesis

Respiratory droplets (containing SARS-CoV-2) and contact transmission are the main routes of infection for COVID-19. It is also known that infected individuals can spread the virus to other humans with virus-containing body fluids such as sputum and saliva through the oral and nasal cavities and possibly other mucous membranes (e.g. the eyes) [3]. However, it is still unclear whether asymptomatic infected individuals can spread the infection. SARS-CoV-2 binds to human lung cells by interaction of the Spike (S) glycoprotein of the virus to its receptor, angiotensin converting enzyme 2 (ACE2), and initiates viral entry into type II pneumocytes [6]. The S protein includes two domains: the S1 domain mediates binding to ACE2, and the S2 domain promotes fusion to host cell membrane [6]. The higher transmission rate of SARS-CoV-2 in comparison to other coronaviruses, such as SARS-CoV and MERS-CoV, (3–10 fold) is believed to be a result of four amino acid changes in the S1 domain [7]. Upon virus entry and infection of pneumocytes, viral pathogen-associated molecular patterns (PAMPs) are recognized by the host pattern recognition receptors (PRRs) which leads to local inflammatory responses and cytokine secretion such as transforming growth factor-β1 (TGF-β1), tumor necrotic factor-α (TNF-α), interleukins 1β and 6 (IL-1β and IL-6) [8]. In severe COVID-19 cases, a cytokine storm, defined as dysregulated and excessive immune responses, is associated with acute respiratory distress syndrome (ARDS) and multiple organ failure [8]. Elevated levels of IL-2, IL-6, IL-7, IL-10, IL-12, interferon-γ (IFN-γ), interferon-induced protein-10 (IP-10) and TNF-α, and lymphopenia are associated with the severity of the disease and fatal outcomes [9]. Humoral immunity plays a key role in the protection against the virus [10]. The production of neutralizing antibodies can be detected as early as four days after the infection [10]. Specific-IgM antibodies peak on the ninth day post-infection, while specific-IgG antibodies are detected after 3 weeks [10]. Cell-mediated immunity also plays an important role in mediating the immune response to SARS-CoV-2 [11]. Antigen-specific T cells such as regulatory CD4+ T cells and CD8+ T cells, balance the battle against the virus and suppress the overproduction of cytokines that lead to aggressive inflammation [12]. In addition, CD4+ helper T cells activate T-dependent B cells and increase the production of neutralizing antibodies; while cytotoxic CD8+ T cells kill virus-infected cells to release the virus for neutralization by antibodies [13].

## Challenges in vaccine development–lessons learned from TB

Characterization of the genetic material and the antigenicity encoded by different genomic regions are important factors for vaccine development. The sequencing of *M. tuberculosis* genome and advances in comparative genomics and bioinformatics have helped to identify species-specific genomic regions and encoded proteins in different mycobacterial species [14, 15]. By using subtractive genomic hybridization to identify genetic differences between the virulent *M. bovis* and *M. tuberculosis*, and the avirulent vaccine strain of *M. bovis* Bacillus Calmette–Guérin (BCG), Mahairas and colleagues, for the first time, had shown the existence of three distinct genomic regions of differences (RDs), designated as RD1 to RD3, which were found to be deleted in BCG [16]. In 1999, by using the whole genome sequence data of *M. tuberculosis* and comparisons with other mycobacterial species, such as *M. bovis* and BCG, it was shown that 16 regions of differences (RD1 to RD16) existed among *M. tuberculosis*, *M. bovis* and BCG [17, 18]. The identification of major *M. tuberculosis*-specific antigens has provided a better understanding in the role of these antigens in inducing protective immunity against TB and expanded the TB vaccine development strategies (Table 1) [19].

**Table 1:** Pipeline of novel TB vaccines in clinical trials

Vaccine name	Vaccine type	Clinical phase	Description
Ad5 Ag85A	Adenovirus-vector	I	BCG booster—Adenovirus vector expressing Ag85A, Ag85B and TB10.4 antigens [41]
MVA85A	Vaccinia Ankara virus-vector	I	BCG booster—Modified vaccinia Ankara virus vector expressing Ag85A [42]
GamTBVac	Subunit vaccine	I	BCG booster—Dextran-binding domain modified Ag85a and ESAT6-CFP10 MTB antigens and CpG ODN adjuvant, formulated with dextrans [43]
AEC/BC02	Subunit vaccine	I	BCG booster—Ag85b, ESAT6-CFP10 combined with BCG-derived CpG and aluminum salt adjuvant [44]
ID193/GLA-SE	Subunit vaccine	IIa	BCG booster—Oil in water emulsion/TRL4 agonist [45]
MTBVAC	Live attenuated	IIa	Live attenuated *M. tuberculosis* strain SO2with mutation in phoP transcription regulator gene [46]
TB/Flu-04L	Influenza-vectored	IIa	Influenza virus strain A/Puetro Rico/8/34 H1N1 expressing Ag85A and ESAT6 [47]
RUTI	Live attenuated	IIa	Therapeutic vaccine-purified and liposomal cellular fragments of *M. tuberculosis* [48]
H56:IC31	Subunit vaccine	IIb	BCG booster—fusion protein of Ag85B, ESAT-6 and Rv2660c formulated in IC31 adjuvant [49]
M72/ASO1_E_	Subunit vaccine	IIb	BCG booster—fusion protein of Mtb32A and Mtb39A combined with AS01 adjuvant [50]
DAR-901	Inactivated whole-cell	IIb	BCG booster—whole cell or extract of *M. obuense* [51]
VPM1002	Live recombinant	III	Pre and post exposure vaccine—live recombinant *M. bovis* expressing listeriolysin of *Listeria monocytogenes*, lacing the urease C gene and containing hygromycin resistance marker [52]
MIP	Heat killed	III	Therapeutic vaccine—whole cell of extract of *M. indicus pranii* [53]
*M. vaccae*	Heat killed	III	Therapeutic vaccine—whole cell of extract of *M. vaccae* [54]

The novel SARS-CoV-2 genome sequence is clearly less complex than that of *M. tuberculosis* and was available to the public only a few weeks after its emergence [20]. The availability of such data made it possible for scientists to develop multiple vaccine candidates targeting different areas of the virus genome [20]. The comparative genomic analysis between the coronaviruses SARS-CoV-2, SARS-CoV, MERS-CoV showed 79% and 50% sequence identity, respectively [21]. In fact, the SARS-CoV-2 showed higher genetic similarity (93.1%) to RaTG12 virus isolated from bats, suggesting its zoonotic origin [20]. However, the most variant amino acid sequence between SARS-CoV-2 and SARS-CoV was found in the Spike (S) protein of the SARS-CoV-2 virus with similarity of 64% in the S1 domain and 90% in the S2 domain [22]. Furthermore, Ju and colleagues identified 206 monoclonal antibodies derived from B cells isolated from COVID-19 patients that bind to the viral S protein receptor-binding domain (RBD) [23]. These antibodies were found to be SARS-CoV-2 specific and did not cross-react with SARS-CoV and MERS-CoV [23]. This approach of identifying specific epitopes and their antigenicity is a crucial step for development of the vaccine.

In order to optimize the efficacy of vaccine formulations, adjuvants and delivery systems are often adopted. Adjuvants enhance the immunogenicity of an antigen in various ways such as (i) stabilization of the antigen and protection from physical and/or chemical degradation in vivo and therefore induce a potent and persistent immune response, (ii) enhancing antigen uptake, (iii) directing the antigen to specific immune cells, (iv) enhancing the stimulation of different T and/or B cells, and (v) enhancing antigen presentation [24, 25]. For TB vaccines, adjuvants and delivery systems that induce and modulate the secretion of protective Th1 immune responses, rather than pathologic Th2 and Treg immune responses, are preferred [24, 26]. The examination of chemical adjuvants, live recombinant bacteria or viruses, and DNA vaccine constructs, in combination with immunodominant TB antigens, have been shown to induce protective Th1 responses and reduce the bacterial loads in the lungs of infected mice [27–30]. There are a number of TB vaccines in the clinical trials pipeline that are being developed as primary (or replacement) vaccines or as secondary boosters to the available TB vaccine, BCG. Vaccinating healthy BCG-vaccinated adults with AERAS-402 (adenovirus expressing a fusion of *M. tuberculosis* antigens 85A, 85B, and TB10.4) and MVA85A (Modified Vaccinia Ankara expressing Ag85A) has been found to be safe, and boosted antigen-specific CD4+ and CD8+ T cell responses in phase I clinical trials [31]. Similarly, vaccinating both BCG-naïve as well as previously BCG-vaccinated adults with a human type 5 adenovirus-based vaccine expressing Ag85A (adHu5Ag85A) was shown to be safe and elicited appropriate T cell responses [32].

The selection of adjuvants that enhance protective immunity against SARS-CoV-2 is crucial. In contrast to TB, neutralizing-antibodies appear to play a major role in protection against the virus [33]. This has led to the use of convalescent plasma from the COVID-19 recovery patients as a treatment strategy has had a positive impact on clinical outcome and therefore suggests a vaccine that can induce the secretion of neutralizing antibodies is ideal [33]. However, the aim of the adjuvant and delivery systems used for SARS-CoV-2 vaccines should not only be limited to antibody production but also aim to produce long-lasting immunity against the virus. Long-lived plasma cells (PC) have been shown to have a critical role in maintaining antibody levels [34]. It is still unclear whether the induction of long-lived PC is a result of vaccinating with multivalent protein antigens, type of adjuvant used, number of boosters, or an ideal combination of them all [34]. In addition, vaccination route can contribute to antibody production. This has been demonstrated for TB for which studies have shown that immunization of mice with BCGintranasally and by aerosols induced the production of IgG and IgA antibodies more than subcutaneous immunization [35].

Although BCG has been used extensively in large parts of the world and over four billion people have been vaccinated since the 1920s, it has failed to show consistent protective efficacy in humans, particularly in the developing world and against adult pulmonary disease, the most common manifestation of TB [4]. Even though neonatal BCG vaccination offers protection in children up to 10–20 years, the efficacy of BCG protection against adult pulmonary TB varies from 0 to 80% [36, 37]. It is thought that this variation is due to different BCG preparations (and possibly different strains), exposure to certain *M. tuberculosis* strains and/or environmental mycobacteria, genetic variations among host populations, climatic and living conditions [15, 36]. In comparison, genetic mutation and nucleotide substitution, which can lead to shifts in protein translation, are amongst the most important mechanisms of viral evolution in nature and impact on vaccine efficacy. Becerra-Flores and Cardozo reported that a mutation at position 614 of the SARS-CoV-2 spike, leading to amino acid change from aspartate to glycine, increased the virus pathogenicity and was associated with higher mortality rates globally [38]. Similarly, host genetic variation may be important, Al-Mulla and his colleagues have studied the genetic variations in *ACE2*, *TMPRESS2*, and *FURIN*, SARS-CoV-2 receptors in humans, in the Middle Eastern (Kuwait, Qatar and Iran) and European populations. They have concluded that the lower mortality rates in the Middle Eastern populations can be explained by the frequent deleterious variants in the FURIN gene in these populations, but not in European populations [39]. Therefore, ideally, clinical trials of vaccine candidates must be examined against different strains of the SARS-CoV-2 virus and within different populations and age groups.

## Current status of novel TB vaccines

Novel TB vaccine candidates aim to either boost or replace the current available vaccine, BCG. Vaccines in development are targeting different stages of the infection/disease and different age groups. TB vaccines can be classified as (1) pre-exposure vaccines targeting individuals who have not been vaccinated with BCG, exposed to mycobacterial antigens, nor infected with TB, typically neonates, (2) post-exposure vaccines targeting individuals vaccinated with BCG, and (3) therapeutic vaccines targeting individuals infected with TB to accelerate the clearance of the pathogen (Table 1) [40].

## Progress in the development of vaccines against COVID-19

To date, more than 230 COVID-19 vaccines are being developed using various technologies [55–63], some of which are “traditional” such as inactivated [62, 63], viral-vector vaccines [58, 60, 61] and adjuvanted subunit vaccines [56]. Other vaccine technologies being developed have not been used in licensed vaccines before e.g. mRNA and DNA vaccines [55, 57, 59]. The leading vaccines in clinical trials are viral-vectored expressing the S protein of the SARS-CoV-2 [58, 60, 61], mRNA vaccines [57, 59], inactivated and adjuvanted vaccines [56, 62, 63] (Table 2). The aim of these vaccines is to protect from the infection and/or prevent clinical symptomatic disease and therefore reduce disease severity. At the time of writing Pfizer have reported in the media promising Phase III results for their mRNA based vaccine, but the data is not yet available.

**Table 2:** Pipeline of major COVID-19 vaccines in clinical trials

Vaccine name	Vaccine type	Clinical phase	Description
INO-4800	DNA	I/II	Synthetic DNA vaccine targeting SARS-CoV S protein [55]
RBD-dimer	Recombinant subunit	II	Beta-CoV vaccine against beta coronaviruses [56]
CTII-nCoV	Adenovirus-vector	II	Adenovirus vector encodes for full-length S protein [58]
BNT162	mRNA	III	Four vaccine candidates include: BNT62a1 and BNT62b1: contain nucleoside modified RNA, BNT162b2: uridine containing mRNA, and BNT162c2: using self-amplifying mRNA. Each is composed of S protein and combined with a lipid nanoparticle formulation [57]
mRNA-1273	mRNA	III	Lipid nanoparticle (LNP)-encapsulated mRNA-based vaccine encoding full length S protein [59]
Ad26COVS1	Adenovirus-vector	III	Replication deficient adenovirus type 5 vector expressing S protein [60]
AZD1222	Adenovirus-vector	III	Chimpanzee adenovirus vector expressing S glycoprotein [61]
CoronaVac	Inactivated virus	III	Adsorbed COVID-19 (inactivated) vaccine [62]
BBIBP-CorV	Inactivated virus	III	Inactivated SARS-CoV-2 HB02 strain [63]

## BCG as a vaccine for COVID-19?

After the introduction of BCG in the 1920s, epidemiological studies have demonstrated that BCG vaccination not only protected against childhood TB, but also had an impact in reducing childhood mortality independently of TB protection [64]. Studies have suggested prophylactic effects of BCG in bacterial and viral infections as well as therapeutic effects in human papillomavirus (HPV) infection and non-invasive bladder cancer [65–67]. The prophylactic and therapeutic effects of BCG may be due to the induction of ‘trained’ immunity by BCG vaccination that protects against unrelated or nonspecific infections [68]. BCG vaccination induces the innate immune cells memory by activating natural killer cells, monocytes, and macrophages and by releasing pro-inflammatory cytokines IL-1β (TGF-β1), TNF-α [68, 69]. In addition, BCG vaccination induces the secretion of various lymphocyte responses which involve the activation of CD4+ and CD8+ memory cells and modulation of Th1 and Th17 responses to non-specific infections [69].

Several ecological studies have observed the relationship between BCG vaccination and COVID-19 and they showed that COVID-19 incidence and/or mortality rates are significantly lower in the countries with universal BCG vaccination policies (e.g. China, Japan, Peru, Chile, Bangladesh) compared to countries without BCG vaccination policies (e.g. Argentina, Spain, Italy, United States) [70, 71]. It is proposed that the low incidence and/or mortality rates in these counties may, in part, be due to the protective role of BCG against viruses and to the fact that there is a degree of homology between amino acid sequences expressed by some BCG strains and SARS-CoV-2 [72]. However, although these findings are very interesting, such comparisons should be treated with caution. National lockdown measurements, population age median and health complications, and other aspects contribute to COVID-19 incidence and mortality rates. In addition, the duration of BCG protection in humans is subject to debate and there is not enough evidence to how long this protection lasts [73]. Therefore, randomised controlled trials are needed to determine the protective role of BCG vaccination against COVID-19. There are several clinical trials exploring the effect of BCG vaccination on COVID-19 [74].

## Possible complications of COVID-19 vaccines

Vaccine safety is crucial in the vaccine development process. A number of health and safety issues can arise from vaccination that can vary from mild fever to death. Even though live attenuated vaccines mount a natural infection and can induce a primary immune response better than inactivated whole-cell and adjuvanted vaccines, there are safety concerns when vaccinating with live attenuated pathogens. This is illustrated by immunization of children with BCG which may lead to various complications such as BCG lymphadenitis, injection site complications, and disseminated BCG disease [75]. In addition, BCG vaccination can cause disseminated BCGosis in immunocompromised individuals, e.g. HIV infected children, and therefore BCG is not approved as a vaccine for HIV-exposed neonates in numerous countries [40, 76]. The impaired T-cell responses in HIV-infected children, low BCG-induced CD8 T cell responses, IFN-γ, TNF-α and IL-2 cytokines secretion, and expression of “low quality” T cell responses, may explain the low protection provided by BCG and the dissemination of BCGosis [77].

Viral-vectored vaccines expressing bacterial/virus specific antigens are considered safer than live attenuated vaccines. However, preexisting antibodies to the viral vector and inadequate human immune response in response to the vectored vaccines are the main limitations. In phase I clinical trials of TB vaccination, the immune response to the TB Ad5 Ag85A vaccine candidate was correlated with preexisting anti-adenovirus antibodies [32]. This limitation may be resolved by changing the route of immunization. Satti and colleagues reported that aerosol administration of TB vaccine candidate MVA85A in humans overcame preexisting antibodies against the vaccine vector, whereas serum antibodies for the viral vector were detected after intradermal administration [78]. In addition, development of modified adenovirus vaccines, such as chimpanzee adenovirus-vectors that can induce cellular and humoral cell responses may be another option [79]. Inadequate immune responses generated by inactivated viral vaccines and/or viral-vectored vaccines can cause an adverse reaction known as antibody-dependent enhancement (ADE) [70]. In ADE, viral entry into host cells is enhanced by efficiency of virus-antibody complex to FcR bearing cells. This is often noticed when the vaccine-induced antibody fails to neutralize the virus because of diluted antibody levels or inaccurate specificity [80]. This can lead to macrophage activation and inflammatory cytokine secretion and subsequently tissue damage [80]. Yip et al. showed that human macrophages were infected by SARS-CoV as a succeeding Ig-G mediated ADE [81]. Additionally, vaccinating with SARS-CoV vaccine candidate expressing spike protein induced the infection human B cells in vitro [82]. The mechanism required to develop a vaccine that mimics the natural infection and induces adequate immune responses remains an ambitious immunological approach. The immunological and serological specifications that can explain the correlation of protection against SARS-CoV-2 infection, especially neutralizing antibody titer, need to be explored.

## Conclusion

Vaccines are important tools to control and eradicate the infectious diseases. The recent SARS-CoV-2 pandemic has overwhelmed the world as there are no vaccines or effective anti-viral agents currently available to control the disease. The vaccines against SARS-CoV-2 are expected to induce both neutralizing antibodies and cellular immunity. The process of identifying appropriate antigens and delivery systems for SARA-CoV-2 vaccine is ongoing and candidate vaccines are being evaluated in experimental animal models and human clinical trials. However, the development of vaccines can be cumbersome and time-consuming as shown by the development pathway for TB. We propose that as a respiratory infection, albeit a bacterium, with a wide spectrum of host susceptibility, TB provides a useful model for COVID-19 vaccine development from which lessons can be learnt.

## Data Availability

Not applicable.
